# First seroprevalence and molecular identification report of *Brucella canis* among dogs in Greater Cairo region and Damietta Governorate of Egypt

**DOI:** 10.14202/vetworld.2023.229-238

**Published:** 2023-01-31

**Authors:** Mahmoud E. R. Hamdy, Mahmoud H. Abdel-Haleem, Rehab E. Dawod, Rania I. Ismail, Soliman S. Hazem, Hanan A. Fahmy, Nour H. Abdel-Hamid

**Affiliations:** 1 Department of Brucellosis Research, Agricultural Research Center, Animal Health Research Institute, P.O. Box 264-Giza, Cairo 12618, Egypt; 2 Department of Bacteriology, Agricultural Research Center, Animal Health Research Institute (Damietta Branch), Egypt; 3 Department of Biotechnology, Animal Health Research Institute, Agricultural Research Center, Dokki, Giza, 12618 Egypt

**Keywords:** 2-mercaptoethanol tube agglutination test, AMOS-polymerase chain reaction, Bruce-ladder polymerase chain reaction, *Brucella* *canis* species-specific-polymerase chain reaction, *Brucella canis*, rapid slide agglutination test

## Abstract

**Background and Aim:**

Given the rise in stray and imported dogs in Egypt over the past 5 years, it is surprising that no report of *Brucella canis* infection in dogs or humans has been documented in Egypt’s published papers. This study aimed to detect the presence of antibodies against the rough (*B. canis*) and smooth *Brucellae* among dogs in Egypt and to characterize the *Brucella* species circulating in dogs.

**Materials and Methods:**

Blood samples (n = 449) were collected from owned and stray dogs in the Greater Cairo region (n = 309) and Damietta governorate (n = 140). The apparent, true, and total seroprevalence of canine brucellosis caused by *B. canis* infection were calculated using the 2-mercaptoethanol tube agglutination test (2-ME TAT) and rapid slide agglutination test (RSAT). We used the rose Bengal test (RBT) and the buffered acidified plate antigen test (BAPAT) to check the serum samples from dogs for the presence of antibodies against smooth *Brucellae*. Three polymerase chain reaction (PCR) assays - Bruce-ladder PCR, *B. canis* species-specific PCR (BcSS-PCR), and Abortus Melitensis Ovis Suis (AMOS)-PCR - were used to determine the *Brucella* species in the buffy coats of the serologically positive dogs.

**Results:**

The overall apparent and true prevalence of *B. canis* infection in dogs were estimated to be 3.8% and 13.2%. The estimated true prevalence in stray dogs (15%) was higher than in owned dogs (12.5%). The BAPAT and the RBT using smooth antigens revealed that 11 (2.4%) and 9 (2%) were positive. Bruce-ladder PCR targeting *eryC, ABC*, and *Polysaccharide deacetylase* genes was able to identify *B. canis* in nine out of 17 buffy coat samples. AMOS-PCR identified the eight undetermined *Brucella* species by Bruce-ladder PCR as *Brucella abortus* (n = 4) and *Brucella melitensis* (n = 4). To exclude the presence of *Brucella suis*, a one-step species-specific BcSS-PCR was performed and specifically amplified all *B. canis* DNA (n = 9) the same as did the Bruce-ladder PCR.

**Conclusion:**

To the best of our knowledge, this is the first report of *B. canis* detection in dogs in Egypt. Molecular identification of *B. abortus* and *B. melitensis* in the Egyptian canines highlights the role of stray dogs in brucellosis remerging in Brucellosis-free dairy farms. *Brucella canis* infection can be diagnosed specifically with the one-step BcSS-PCR. The obtained results set-an-alarm to the veterinary authorities to launch plans to control this disease in dogs.

## Introduction

The genus *Brucella* comprises 12 species, six of them are classical species, including; *Brucella abortus, Brucella melitensis, Brucella suis, Brucella ovis, Brucella canis*, and *Brucella neotomae* [1, 2], and six recently isolated species, namely; *Brucella ceti, Brucella pinnipedialis, Brucella microti, Brucella inopinata*, *Brucella papionis*, and *Brucella vulpis* [3, 4]. Among all of the *Brucella* members, four species are most commonly involved in causing the disease in humans, namely; *B. melitensis, B. suis, B. abortus*, and *B. canis*. *Brucella canis* is a highly infectious pathogen that infects primarily dogs, leading to reproductive disorders and abortion. Despite the increasing international movement of dogs, there might be a growing risk of *B*. *canis* as a member of the genus *Brucella*. The *Brucella* organism causing canine brucellosis was first isolated by Carmichael in 1966 in the USA from aborted beagles and was termed *B. canis* [5]. Although *B. canis* is a rough organism, it is still pathogenic to canines and humans. The first report on *B. canis* infection in humans was in 1968 by the national Centers for Disease Control and Prevention of the US Public Health Service, by a laboratory technician who handled viable organisms. *Brucella canis* infection in female dogs is often linked to reproductive problems leading to infertility, abortion, and endometritis, even though many instances are clinically mild. However, a wide range of non-reproductive conditions, such as discospondylitis, endophthalmitis, and chronic uveitis, can also manifest [6]. Along with non-specific clinical symptoms such as fatigue, decreased appetite, and weight loss, lymphadenitis is another common condition in both male and female dogs [7]. Dogs infected with *B. canis* displayed prostate gland inflammation, epididymitis, orchitis, and scrotal edema [6]. The *B. canis* transmission between male and female dogs during mating periods is proven to disseminate organisms in seminal fluid leading to venereal transmission [8]. Besides, *B. canis* could be shed in the infected dog’s urine, blood, and saliva. *Brucella canis* infection in dogs occurs most commonly from ingestion of vaginal discharges contaminated with huge numbers of organisms after an abortion [8]. Once these canines are infected, the infection is either self-eliminated within 2–3 years or develops into a lifelong infection [7]. The antibiotics used to treat *B. canis* infections are mostly ineffective [7].

Human infections with *B. canis* are relatively mild compared to those caused by other *Brucella* species [9]. However, all clinical features of Brucellosis in humans due to infection with *B. canis* are similar to those induced by other pathogenic *Brucella* species. The disease is transmitted to humans through direct and indirect transmission and is considered a classical debilitating zoonosis causing undulant fever [10]. The rapid slide agglutination test (RSAT) and the 2-mercaptoethanol tube agglutination test (2-ME TAT) are the serological methods most frequently used to diagnose *B. canis* in dogs [1]. These serological tests have limitations of low sensitivity and variable specificity [11]. Bacteriological examination of the causative agent through blood culture is usually applied to isolate *B. canis*. However, bacteremia is frequently intermittent or absent in chronic infections. Due to these shortcomings, different molecular techniques have been developed for the rapid and direct detection of *B. canis* as real-time polymerase chain reaction (PCR), Bruce ladder multiplex PCR, and *B. canis* species-specific (BcSS) PCR [12–14]. The world population of dogs is estimated at around 900 million [15]. The estimated stray dogs population in Egypt is about 15 million [16]. Dogs that are held by owners are registered and identified by collars or micro-chips, while unowned dogs include stray or guarding animal-farms dogs are not identified or registered or controlled. *Brucella melitensis* bv3 and, less frequently, *B. abortus* bv 1 are the predominant *Brucella* strains circulating in livestock in Egypt, according to a number of studies used to determine the prevalence of various *Brucella* strains in domestic farm animals [17, 18]. Only two studies accidentally isolated *B. abortus* from two stray female dogs roaming on infected dairy cattle farms [19, 20]. To the best of our knowledge, *B. canis* has not been reported in dogs or humans in Egypt in any published report.

This study aimed to detect the evidence of *B. canis* antibodies in dogs’ sera in two areas for the first time in Egypt and to molecularly identify the *Brucella* DNA from the blood buffy coat of infected canines.

## Materials and Methods

### Ethical approval

The Animal Health Research Institute’s Research Ethics Committee for Experimental and Clinical Studies approved the study protocol, which adhered to the guidelines of the World Health Organization and the Helsinki Declaration.

### Study period and location

The study was conducted from June 2019 to January 2022. Blood samples were collected from randomly selected adult dogs (n = 449) in two different regions in Egypt. The first one was the Greater Cairo region (owned dogs) which includes the following; all cities in Cairo Governorate as well as Giza, 6^th^ of October city, Sheikh Zayed City in the Giza Governorate, Shubra El Kheima, and Obour in the Qalyubia Governorate with the aid of personalized dog owners. The second one was the Damietta Governorate (stray dogs) from Damietta city, Faraskur city, and Kafr Saad. Serum samples were separated from the blood, divided into aliquots, and preserved at ‒80°C until being examined. The reason why these areas were selected is that the greater Cairo Province is the largest metropolitan area in Egypt, the largest urban area in Africa, the Middle East, and the 6^th^ largest metropolitan area in the world, with a total population estimated at 20,901,000; area: 1,709 km^2^; density: 10,400/km^2^ [21]. While Damietta governorate is famous for having dairy farms where stray dogs cross the fences of these farms.

### Experimental design

#### Study design and sampling

The sample size was estimated using the online WinEpi tool [22]. An estimated sample size of 449 blood samples with and without sodium citrate was collected from owned dogs in the Greater Cairo region (n = 309) and from stray dogs located in Damietta governorate (n = 140). These samples were tested for the detection of *Brucella* antibodies (RSAT and 2-ME TAT) and *Brucella* DNA by three PCR assays for the detection of smooth and rough *Brucellae* circulating in the blood of dogs in the study area.

#### Estimation of seroprevalence

Using the online WinEpi tool [22], we estimated the minimum sample size to be 385 dogs. This was calculated based on the population size of 15 million dogs, a confidence level of 95%, an accepted type 1 error of 5%, and a minimum expected prevalence of 50% (First report of *B. canis* prevalence estimation in Egypt). The estimated sample size was adjusted to be 449 blood samples.

The apparent prevalence (AP) of canine brucellosis caused by *B. canis* infection was calculated by dividing the total number of seropositive dogs identified by both RSAT and 2-ME TAT by the total number of tested dogs. We calculated the true prevalence of canine brucellosis caused by *B. canis* using the formula TP = (AP+CSp-1)/(CSe+CSp-1) [23, 24] using the combined sensitivity (CSe) and combined specificity (CSp) of RSAT and 2-ME TAT. Based on the reported sensitivities and specificities by Keid *et al*. [25], the CSe of both RSAT and 2-ME TAT was calculated [22] to be in series CSe of 28.1% and CSp of 99.9%.

### Tests and procedures

#### Serological tests

The following serological tests were used to check the serum samples from the dogs for the presence of antibodies specific to the rough and smooth *Brucellae*: The RSAT, the 2-ME TAT, rose Bengal test (RBT), and the buffered acidified plate antigen test (BAPAT). DNA of *Brucella canis* and other smooth *Brucellae* were identified in buffy coat samples separated from whole blood collected from seropositive dogs by three types of conventional PCRs (AMOS-PCR, Bruce-ladder PCR, and BcSS-PCR).

*Brucella* canis rough antigen that was used in the RSAT and 2-ME TAT was prepared from heat-killed cells of *B. canis* reference strain RM666 suspended in a formalized phosphate-buffered saline solution. *Brucella canis* 2-ME TAT antigen was obtained from the National Veterinary Services Laboratory (NVSL) Ames IA 50010, USA. Both RSAT and 2-ME TAT were carried out in this study to detect antibodies against *B. canis*, according to the technique adopted by Alton *et al*. [1].

RBT and BAPAT originating from smooth *Brucella* *abortus* antigens were performed in this study to test dog blood samples for the detection of smooth *Brucella* antibodies according to the techniques described by Alton *et al*. [1] and World Organization for Animal Health (WOAH) [2].

### Polymerase chain reaction assays

A. DNA Purification from Buffy Coat Samples with the Higher Purity™ Blood Genomic DNA Extraction Kit (Canvax Biotech, Spain).

Buffy coats fractions were separated after centrifuging 5 mL of citrated whole blood at 2500 × g for 10 minutes at room temperature (15°C–25°C), then transferred into a 15 mL tube containing 1500 μL of S1 buffer. This process is followed by gently mixing and centrifugation at 2000× g for 5 min. The supernatant was removed and the pellet was treated with proteinase K (250 μL) and S2 buffer (5 mL), incubated for 1 h at 55°C in a water bath, and then allowed to cool at 15°C–25°C. The S3 buffer was then added, followed by centrifugation. The supernatant was then gently mixed and transferred to a fresh 15 mL tube containing 5 mL isopropanol. The process involved centrifuging the sample, removing the supernatant using a pipette, and drying the pellet. The tube was centrifuged at 2000 × g for one minute after the pellet had been washed with 5 mL of 70% ethanol. In addition, we dried the pellet after removing the supernatant with a pipette. We added 500 μL of Buffer EB to the pellet, mixed it for 5 s at medium speed in the vortex, and then allowed it to incubate for 1 h. The eluted DNA was stored at −20°C.

### Bruce-ladder PCR [26]

#### Oligonucleotide primer

Primers supplied by Eurofins Scientific (USA) and the cyclic conditions are listed in Table-1 [25].

**Table-1:** Primers sequences, target genes, amplicon sizes, and cycling conditions of Bruce-ladder PCR.

Target gene	Primers sequences	Amplified segment (bp)	Primary denaturation	Amplification (35 cycles)			Final extension	Reference

				Secondary denaturation	Annealing	Extension		
*Polysaccharide deacetylase*	BMEI1436f ACG CAG ACG ACC TTC GGT AT	794	95°C 7 min	95°C 35 s	64°C 45 s	72°C 3 min	72°C 6 min	[25]
	BMEI1435r TTT ATC CAT CGC CCT GTC AC							
*eryC*	BMEII0428f GCC GCT ATT ATG TGG ACT GG	587						
	BMEII0428r AAT GAC TTC ACG GTC GTT CG							
*ABC transporter binding protein*	BR0953f GGA ACA CTA CGC CAC CTT GT BR0953r GAT GGA GCA AAC GCT GAA G	272						

A. Fifty µL reaction containing 25 µL of Emerald-Amp Max PCR Master Mix (Takara, Japan), 1 µL of each primer at a concentration of 20 pmol (for a total of 6 µL of used primers), 14 µL of water, and 5 µL of DNA template was used to utilize the primers. Thermal cycler 2720 from Applied Biosystems (USA) was used to perform the reaction.

B. Oligonucleotide primers and cyclic conditions for AMOS-PCR and BcSS PCR (BcSS-PCR. Primers used were supplied from Metabion (Germany) and listed in Table-2 [14, 27].

**Table-2:** Primers sequences, target genes, amplicon sizes, and cycling conditions of duplix AMOS-PCR and BcSS-PCR.

Target gene	Target agent	Primers sequences	Amplified segment (bp)	Primary denaturation	Amplification (35 cycles)			Final extension	Reference

					Secondary denaturation	Annealing	Extension		
IS711	*B. abortus*	1S711-specificPrimer TGC-CGA-TCA-CTT-AAG-GGC-CTT-CAT *B. abortus*-specific Primer GAC-GAA-CGG-AAT-TTT-TCC-AAT-CCC	498	94°C 5 min	94°C 30 s	55°C 40 s	72°C 45 s	72°C 10 min	[27]
	*B. melitensis*	1S711-specificPrimer TGC-CGA-TCA-CTT-AAG-GGC-CTT-CAT *B. melitensis*-specific Primer AAA-TCG-CGT-CCT-TGC-TGG-TCT-GA	731	94°C 5 min	94°C 30 s	55°C 40 s	72°C 45 s	72°C 10 min	
BCAN B0548–0549 region in chromosome II of *Brucella canis*	*Brucella canis*	Forward 5-CCAGATAGACCTCTCTGGA-3 Reverse 5-TGGCCTTTTCTGATCTGTTCTT-3	300	94°C 7 min	94°C 35 s	59°C 40 s	72°C 35 s	72°C 10 min	[14]

C. PCR amplification.

• C.1. AMOS-PCR [27]

A 25 µL reaction containing 12.5 µL of Emerald-Amp Max PCR Master Mix (Takara, Japan), 1 µL of each primer at a concentration of 20 pmol (for a total of 3 µL of used primers), 4.5 µL of water, and 5 µL of DNA template were used to test the primers. Thermal cycler 2720 from Applied Biosystems was used to carry out the reaction.

• C.2. BcSS-PCR [14]

Primers were utilized in a 25 µL reaction containing 12.5 µL of Emerald-Amp Max PCR Master Mix (Takara, Japan), 1 µL of each primer of 20 pmol concentration (total 2 µL of used primers), 5.5 µL of water, and 5 µL of DNA template. The reaction was performed in an Applied Biosystems 2720 thermal cycler.

D. Analysis of the PCR Products of Bruce-ladder PCR, AMOS-PCR, and BcSS-PCR.

The products of PCR were separated by electrophoresis on 1% agarose gel (Applichem, Germany, GmbH) in 1× TBE buffer at 15°C–25°C using gradients of 5V/cm. For gel analysis, 40 µL of the Bruce-ladder PCR products were loaded into each gel slot. Whereas after the BcSS-PCR and the AMOS-PCR, each gel slot was loaded with 15 µL of the DNA products. The Gene-ruler 100 bp DNA ladder (Fermentas, Thermo, Germany) was used to determine the fragment sizes. The gel was photographed by the gel documentation system (Alpha Innotech, Biometra), and the data were analyzed through computer software.

## Results and Discussion

Diagnosis of canine brucellosis is primarily based on serological examinations. However, infected dogs with *B. canis* usually give negative results by conventional serological tests based on smooth *B*. *abortus* antigen. This is because rough antibodies to *B. canis* do not cross-react with those induced by smooth species. For that, using a rough antigen obtained from *B. canis* is required to assess the immunoglobulin induced by *B. canis*.

The results of the RSAT and the 2-ME TAT performed on serum samples (n = 449) of the owned and stray dogs in the Greater Cairo province and Damietta governorate were 6.2% (28/449) and 3.8% (17/449) as presented in Table-3. Dogs were interpreted as positive when they gave positive reactions in both RSAT and 2-ME TAT (titers ≥ 1/150). These tests detect antibodies against surface antigens of *Brucella* spp., particularly antibodies against rough lipopolysaccharide (LPS). RSAT is a screening highly sensitive test that may give false positive results due to cross-reaction with other bacteria such as *Pseudomonas, Bordetella bronchiseptica, Salmonella*, *Yersinia enterocolitica*, and *Escherichia coli* [11, 28]. In addition, the test can be negative early in the first 3–8 weeks post-infection [29]. The 2-ME TAT is more specific than RSAT as the 2-mercaptoethanol (2ME) acts as a disulfide bond reducing agent of the immunoglobulin (Ig)M, the class of immunoglobulin responsible for the cross-reaction with Gram-negative bacteria through splitting the disulfide bonds and depolymerizes the IgM pentamer. Therefore, any remaining agglutinating activity after 2ME treatment is due to 2ME-resistant IgG, which is the long-lasting specific class of immunoglobulins in *B. canis* infection [1].

**Table-3:** Serological results of canine serum samples by rough *Brucella canis* antigen, smooth *Brucella* antigens, and Reaction to PCR.

Area	Sex		Reactors to *Brucella canis* rough Ag		Reactors to smooth *Brucella* Ag		Reactors to PCR		
		
			RSAT	2-ME TAT	BAPAT	RBT	Number of identified *Brucella* DNA in the whole blood buffy coat samples	*Brucella canis*	Other *Brucella* species
G. Cairo	Male (n=102)	Owned dogs	6	3	1	1	3	2	*B. melitensis* (n=1)
	Female (n=207)		13	8	3	2	8	5	*B. melitensis* (n=2) and *B. abortus* (n=1)
Total	309		19	11	4	3	11	7	4
%			6.1%	3.6%	1.3%	1%			
Damietta Governorate	Male (n=40)	Stray dogs	3	2	2	2	2	1	*B. melitensis* (n=1)
	Female (n=100)		6	4	5	4	4	1	*B. abortus* (n=3)
Total	140		9	6	7	6	6	2	4
%			6.4%	4.3%	5%	4.3%			
Total Samples	449		28	17	11	9	17	9	8
Percent			6.2%	3.8%	2.4%	2%			

*B. melitensis=Brucella melitensis*, *B. abortus=Brucella abortus*, PCR=Polymerase chain reaction, RSAT=Rapid slide agglutination test, 2-ME TAT=2-mercaptoethanol tube agglutination test, BAPAT=Buffered acidified plate antigen test, RBT=Rose Bengal test, Ag=Antigen

The 2-ME TAT is considered a semi-quantitative test that is employed to confirm the results given by RSAT [30]. Serological diagnosis of canine brucellosis due to infection by *B. canis* is challenging. A combination of different serological tests is required for proper diagnosis. To avoid the false positive results, the buffy coats separated from the whole blood of the same seropositive dogs for *B. canis* by both RSAT and 2-ME TAT were further confirmed by molecular tools.

The calculated apparent prevalence of *B. canis* infection in owned dogs in Greater Cairo province was 11/309 (3.6%), while in stray dogs obtained from Damietta governorate was 6/140 (4.3%). The estimated true prevalence (Table-4) in both owned and stray dogs after including the errors represented in the combined sensitivities and specificities of RSAT and 2-ME TAT in the formula was 12.5% in owned dogs and 15% in stray dogs. The overall apparent and true prevalence were estimated to be 3.8% and 13.2%. Our results match the results of Daly *et al*. [31], who reported an overall apparent *B. canis* seroprevalence of 6.8% and adjusted the estimated true prevalence of 29.4% when they performed in-clinic screening of 3898 dogs over more than 4 years in two South Dakota Indian reservations. The results are also in line with Whitten *et al*. [32], who estimated the apparent seroprevalence of *B. canis* antibodies in dogs entering a Minnesota humane society during 1 year (2016–2017) to be 3.1% (22/943) and 3.5% (8/943) among stray and owner-surrendered dogs by using RSAT.

**Table-4:** Prevalence of canine brucellosis due to *Brucella canis* infection in owned and stray dogs.

Owned dogs	
Apparent Prevalence	3.6%
True Prevalence	12.5%
Stray dogs	
Apparent Prevalence	4.3%
True Prevalence	15%
Overall apparent prevalence	3.8%
Overall true prevalence	13.2%

It is noteworthy to mention that the Egyptian government stopped random killing of stray dogs. Based on this fact, the number of stray dogs increased over the past 5 years. The true prevalence of canine brucellosis due to *B. canis* infection in stray dogs was found to be higher than in owned dogs in this study due to the absence of mating control in stray dogs compared to owned dogs [33]. Stray dogs are considered one of the main reasons for *B. canis* spreading in some European countries [34]. To the best of our knowledge, this is the first study to document *B. canis* in the Egyptian canine population.

On the other hand, the application of the BAPAT and the RBT on 449 canine sera revealed that 11 (2.4%) and 9 (2%) were positive. The BAPAT detected two positive samples more than RBT. Enhancing sensitivity was attributed to the final packed cell volume in the case of BAPA was that of 3%, whereas that of RBT was (4%), and the final pH following the addition of serum in BAPAT was (4.02), and (3.8) in RBT [1]. The main factors that cause the BAPAT to be a little more sensitive than the RBT are the reduced final packed cell volume of BAPA compared to RBT and BAPA’s slightly lower final acidic pH [1]. The presence of smooth LPS detected by serological tests based on smooth *B. abortus* antigen of the BAPAT and RBT indicates infection of these dogs with *Brucella* species other than *B. canis*. However, in a previous study [35], using conventional serological tests targeting smooth *Brucella* antibodies, 6.48% of dogs were positive to smooth *Brucellae*. This relatively high positive percentage of smooth *Brucella* antibodies in dogs in such a study was because most dogs were located in and around the infected dairy farms during the outbreak.

Although the definitive confirmation of infection through the isolation of the causative agent is the gold standard for diagnosis [2], this procedure has low sensitivity, takes a long time, poses a biological hazard, especially for lab workers, and necessitates a BSL3 lab facility. Therefore, using molecular tools to detect causative agents increased the possibility of rapid and accurate detection of *Brucella* species.

We used buffy coats in this study instead of whole blood samples for DNA extraction as buffy coat yields more DNA than an equivalent volume of whole blood [36]. In addition, using the buffy coat in the BcSS PCR assay resulted in approximately 100 times higher sensitivity for *B. canis* direct detection if compared with whole blood [13]. Besides, whole blood contains PCR inhibitors such as heparin, hemoglobin that affects the DNA polymerase activity and decreases the amplification efficiency, IgG that binds to single-stranded genomic DNA, and the presence of antimicrobial drug residues [37].

Bruce-ladder is primarily designated to identify and differentiate all standard *Brucella* species, including *B. abortus, B. melitensis, B. suis*, *B. canis*, *B. ovis*, and *B. neotomae* as well as B*rucellae* in marine mammals and the S19, RB51, and Rev.1 vaccine strains using a total of eight pairs of primers in a single step multiplex PCR [26]. To fit the purpose of the application of Bruce-ladder in identifying and differentiating expected *Brucella*e that may infect canines, three pairs of primers were selected to be used in the current study.

All positive canine blood samples for *B. canis* to rough antibodies (n = 17), and all those positive to smooth *Brucella* antibodies (n = 9) were subjected to DNA extraction from buffy coat and PCR analysis. Out of the 26 buffy coat samples subjected to DNA extraction, all reacting canine blood samples for *B. canis* to rough antibodies (n = 17), as well as those reacting to smooth *Brucella* antibodies (n = 9) were subjected to DNA extraction from buffy coat and prepared for PCR analysis. All 26 samples yielded DNA from the buffy coat fraction of the whole blood. However, nine samples gave negative results by Bruce-ladder PCR. This finding may be attributed to false positive results that may be given by serological test to the other Gram-negative bacteria sharing *B. canis* in antigenicity [6].

**Figure-1:**
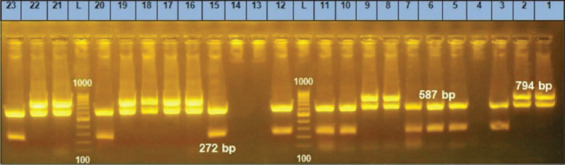
Detection of bands specific for *Brucella* species in the serum samples of dogs by Bruce-ladder polymerase chain reaction. lane 1, *Brucella abortus* reference strain 544; lane 2, *Brucella melitensis* reference strain 16M; lane 3, *Brucella canis* reference strain RM 666; lane 4, Negative control, lanes (5, 6, 7, 10, 11, 12, 15, 20, 23) specific DNA bands for *B. canis*; lanes (8, 9, 16, 17, 18, 19, 21, 22), Specific DNA bands for *Brucella* species other than *B. canis*; lanes (13, 14) negative samples.

To detect different *Brucella* species DNA in canine blood, we investigated the Erythritol Catabolism gene (eryC) existing in all *Brucella* species except *B. abortus* S19 vaccine strain due to the deletion of the 702 bp in BMEI10427-BMEI10428 in this vaccinal strain. Since *B. abortus* S19 is not used or recommended for dog vaccination, the presence of the eryC gene in canine blood refers to any other species or biovars belonging to the genus *Brucella*. The *eryC* gene was also detected in *B. abortus* reference strain 544, *B. melitensis* reference strain 16M, and *B. canis* reference strain RM 666 (Figure-1). Out of 26 positive serum samples for rough and smooth *Brucella* antibodies, the DNA extracted from 17 buffy coat samples were amplified by Bruce-ladder and gave an amplicon size of 587 bp.

Using specific primers (BR0953f and BR0953r), it was attempted to recognize the *ABC* gene, which is responsible for the transporter binding protein, in *B. canis* DNA extracted from the buffy coats of canine blood samples. This resulted in an amplicon size of 272 bp of nine samples and only in the positive control of *B. canis* reference strain RM 666.

Bruce-ladder PCR was employed using primers (BMEI1436f and BMEI1435r) targeting *Polysaccharide deacetylase* present in all *Brucella* species, including all members of *B. abortus* and *B. melitensis* in an amplicon size of 794 bp, which does not exist in *B. canis*. The absence of the 794-bp fragment distinguished *B. canis* from other species [26] and confirmed the presence of *B. canis* DNA in the buffy coat samples. Using the Bruce-ladder PCR targeting the Polysaccharide deacetylase gene to amplify *Brucella* DNA in the given samples (n = 19), Bruce-ladder PCR gave a specific DNA band (794 bp) in eight DNA samples (S8, S9, S16, S17, S18, S19, S21, and S22) as shown in Figure-1. The results of the Bruce-ladder PCR suggest that there are additional, as yet unidentified *Brucella* species (n = 8) other than *B. canis*. In order to identify these eight *Brucella* species, duplex AMOS-PCR was employed.

**Figure-2:**
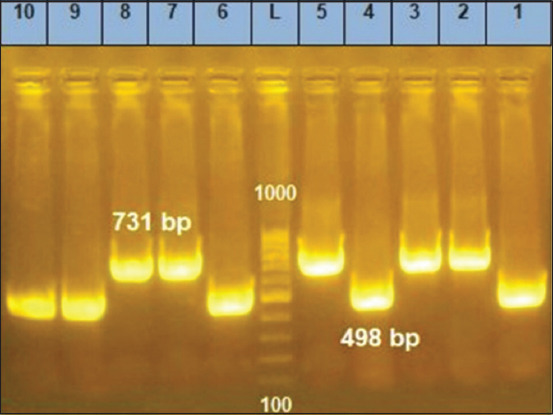
differentiation of unidentified *Brucella* species by Bruce-ladder multiplex polymerase chain reaction (PCR) in the dogs’ serum samples using AMOS-PCR. Lane 1, *Brucella abortus* reference strain 544; lane 2, *Brucella melitensis* reference strain 16M; Lanes 3, 5, 7, and 8 revealed DNA Band (731 bp) specific for *B. melitensis*. Lanes 4, 6, 9, and 10 showed DNA Band specific (498 bp) for *B. abortus*.

Application of Duplex AMOS-PCR [8] on eight samples targeting insertion sequence *IS711*, using *B. abortus* and *B. melitensis* specific primers revealed that four samples’ numbers (S4, S6, S9, and S10) were identified as *B. abortus* with an amplicon size of 498 bp. The other four samples, namely; S3, S5, S7, and S8, showed a specific band at an amplicon size of 731 bp, indicating *B. melitensis* species (Figure-2).

**Figure-3:**
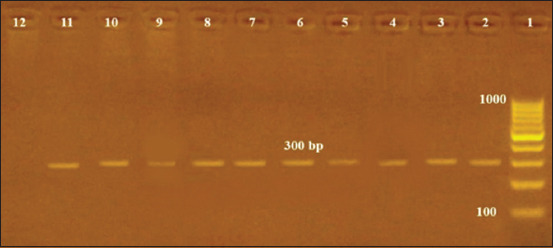
*Brucella canis* species-specific polymerase chain reaction assay used to identify *B. canis* genomic DNA in dogs’ sera. Lane 1, 100 kb DNA ladder; Lane 2, *B. canis* reference strain RM 666; Lanes (3, 4, 5, 6, 7, 8, 9, 10, 11) revealed DNA bands (300 bp) specific for *B. canis*. Lane 12, Negative control.

Given into consideration that dogs’ blood samples were collected from an area far away from pig farms and that those dogs have no history of contact with swine. Since the 272-bp DNA fragment amplified by the Bruce-ladder multiplex PCR is not restricted to *B. canis*. Furthermore, a 272-bp DNA fragment is presented in both *B. suis* and *B. neotomae* [26]. We tried BcSS-PCR assay, a one-step PCR assay (Figure-3), for the detection of *B. canis* samples positive by the Bruce-ladder multiplex PCR to make sure that there was no space for doubt. Kang *et al*. [14] created this sensitive assay, which specifically amplified all *B. canis* DNA extracted from the buffy coat of the whole-blood samples in a 300 bp band. The BCAN B0548-0549 area on *B. canis* chromosome II served as the basis for the creation of the specific PCR primer set employed in this experiment.

We choose the one-step BcSS-PCR for the following reasons: (1) The detection limit of the BcSS-PCR is slightly lower than that of the 16S rRNA PCR and equal to the sensitivity of the BCSP31 PCR using primer pairs of B4/B5 [14]. (2) Using a well-designated PCR primer based on the BCAN B0548-0549 region in chromosome II of *B. canis*, it gives a 300-bp product that is not present in other *Brucella* species or genetically or serologically related bacteria [14]. (3) In addition, the PCR assay using JPF/JPR primer pairs of a gene coding for outer membrane protein (omp-2) that is employed to detect *Brucella* species demonstrated lower sensitivity than the BcSS-PCR [38].

In a previous study, four *Brucella* genes were evaluated by conventional PCR to identify *B. canis* DNA in canine blood, urine, and uterine discharges, including; the gene coding for the BCSP31 protein (primers B4 and B5), the ribosomal gene coding for the 16S-23S intergenic spacer region (primers ITS66 and ITS279), the gene coding for porins (primers JPF and JPRca), and the gene coding for the insertion sequence IS711 (primers O1 and O2) [39]. The gene coding for the 16S-23S rDNA intergenic spacer region was the one that best detected *Brucella* spp. in canine clinical samples, but the 16S rRNA gene PCR assay gave false-positive results for the field strains of *Ochrobactrum anthropi* and *Staphylococcus aureus* [40, 41] while the primers that amplify the insertion sequence IS711 were the most specific one compared with the other primers used in their study with the Sp: 99.66% (confidence interval 98.84–100) [39].

In Egypt, *Brucella* *abortus* was isolated from an apparently healthy Ballady (native) stray bitch roaming in an infected dairy cattle farm in Damietta governorate [20]. Nevertheless, while *B. canis* is the most common cause of canine brucellosis [42], occasional infections with *B. melitensis*, *B. abortus*, or *B. suis* may occur in dogs that have close contact with tissues or secretions of infected livestock animals, especially raw milk, aborted fetuses, and placentas [43]. Multiplex PCR Bruce-ladder assay was recommended by the WOAH as a quick and easy test for molecular identification and typing of more isolated *Brucella* species [2]. Buffy coat samples were used in this study as a source DNA that was amplified by multiplex PCR for detection and identification of *B. canis* was found practical, appropriate, and fit matrix for the purpose of *B. canis* diagnosis in dogs.

*Brucella canis* antibodies were detected in the two dogs’ serum samples by RSAT and 2-ME TAT (S13 and S14). The DNA extracted from the buffy coat of these two serologically positive samples gave negative results to Bruce-ladder PCR (Figure-1). This may be explained by the fact that only a small number of *B. canis* cells, which produce little, undetectable DNA, might be present in the whole blood of these negative samples. This issue could be solved by pooling blood samples from the same suspect dog.

Although the obtained results exhibit evidence of the presence of *B. canis* among dogs in Egypt, the source of this *Brucella* species remains unknown. It might have originated and circulating in Egypt already for many decades or it might be of exotic origin and introduced to the country through crossing international borders with travelers with pet animal companions. Further studies should be conducted to isolate the organism and run WG sequencing to assess the phylogenetic relatedness of this strain with other international strains. Limitations of the present study are that the results refer to random samples dogs collected from dogs in Greater Cairo region and Damietta Governorate and do not reflect countrywide.

## Conclusion

This article reports the presence of *B. canis* in Egypt for the first time and highlights this neglected zoonotic disease. For the first step of diagnosis, serological tests originating from rough *B. canis* antigen, particularly RSAT as a screening test and 2-ME TAT as a supplementary test, followed by PCR assays, was necessary for the accurate diagnosis. The alternative use of multiplex PCR Bruce-ladder assay using only three pairs of primers, followed by AMOS-PCR assay, has proved applicable for the identification of *B. canis* and other *Brucella* species in infected dogs. The one-step BcSS-PCR could be used as a single alternative, accurate, and valuable tool in the diagnosis of *B. canis* infection in the buffy coat samples. Detection of *B. abortus* and *B. melitensis* in canine blood revealed the role of stray dogs in remerging the disease in Brucellosis-free dairy farms in Egypt. The obtained results set-an-alarm to the veterinary authorities to launch plans to control this disease in dogs. One Health approach should be initiated for canine brucellosis interface with humans who come in contact with infected dogs.

## Authors’ Contributions

MERH and NHA: Conceptualization. MERH, NHA, RII, MHA, SSH, RED, and HAF: Methodology. MERH and NHA: Data analysis. MERH and NHA: Data visualization. MERH, and NHA: Data curation. MERH and NHA: Manuscript draft writing. MERH, NHA, RII, MHA, SSH, RED, and HAF: Manuscript review and editing. All authors have agreed to publish the manuscript in its current format. All authors have read and approved the final manuscript.
